# Association of germline genetic variants with *TMPRSS2-ERG* fusion status in prostate cancer

**DOI:** 10.18632/oncotarget.27534

**Published:** 2020-04-14

**Authors:** Indu Kohaar, Qiyuan Li, Yongmei Chen, Lakshmi Ravindranath, Denise Young, Amina Ali, Isabell A. Sesterhenn, Inger L. Rosner, Jennifer Cullen, Shiv Srivastava, Matthew Freedman, Gyorgy Petrovics

**Affiliations:** ^1^Center for Prostate Disease Research, Department of Surgery, Uniformed Services University of the Health Sciences and the Walter Reed National Military Medical Center, Bethesda, Maryland, USA; ^2^Henry Jackson Foundation for the Advancement of Military Medicine (HJF), Bethesda, Maryland, USA; ^3^Department of Medicine, Harvard Medical School and Dana-Farber Cancer Institute, Massachusetts General Hospital, Boston, Massachusetts, USA; ^4^Joint Pathology Center, Silver Spring, Maryland, USA

**Keywords:** prostate cancer, *TMPRSS2-ERG*, molecular subtype, ERG fusion, SNP

## Abstract

Introduction: Oncogenic activation of ERG resulting from *TMPRSS2-ERG* gene fusion is a key molecular genetic alteration in prostate cancer (CaP). The frequency of ERG fusion is variable by race; however, there are limited data available on germline polymorphisms associating with ERG fusion status. The goal of this study is to identify the inherited risk variants associating with ERG status of CaP.

Materials and Methods: SNP genotyping was performed on the Illumina platform using Infinium Oncoarray SNP chip on blood derived genomic DNA samples from 400 patients treated by radical prostatectomy at a single military institution. ERG status was determined in whole mounted prostate specimens by immuno-histochemistry (IHC) for ERG protein expression. Data analysis approaches included association analyses based on EMMAX and imputation by IMPUTE2. Imputed SNPs were validated by ddPCR.

Results: SNP genotyping analysis using imputation identified rs34349373 (p 4.68 × 10^**-8**^) and rs2055272 (p 5.62 × 10^-8^) in *TBC1D22B* to be significantly associated with ERG fusion status in index tumor and non-index tumor foci. Imputed SNP rs2055272 was further experimentally validated by ddPCR with 98.04% (100/102) concordance. Initial discovery analysis based on SNPs on Oncoarray SNP chip, showed significant (p 10^-5^) association for SNPs (rs6698333, rs1889877, rs3798999, rs10215144, rs3818136, rs9380660 and rs1792695) with ERG fusion status. The study also replicated two previously known ERG fusion associated SNPs (rs11704416 in chromsome 22; rs16901979 in chromosome 8).

Conclusions: This study identified SNPs associated with ERG status of CaP.

Impact: The findings may contribute towards defining the underlying genetics of ERG positive and ERG negative CaP patients.

## INTRODUCTION

Prostate cancer (CaP) is a major cause of morbidity and mortality worldwide [1]. It is the second most frequent cancer and the fifth leading cause of cancer death in men [1–3]. In the United States, CaP is the most prevalent non-skin male cancer and ranks second in cancer-related deaths [3]. Oncogenic activation of ERG resulting from prevalent gene fusions (predominantly as *TMPRSS2-ERG*) is a key driver event in CaP pathogenesis [4–6]. Multiple studies have reported a significantly lower frequency of ERG positivity in CaP tumors of African American (AA; 23%) compared to Caucasian American (CA; 49%) patients [4, 7–9]. A significant correlation between ERG negativity in CaP tumors with development of distant metastasis, as well as biochemical recurrence (BCR) was noted in CA men by comprehensive analysis on 930 whole mount prostate specimens in AA and CA men [7]. Additionally, AA patients with high Gleason grade tumors [8–10] exhibited primarily ERG negative index tumor type [8, 10]. However, there are also controversial reports on the association of ERG fusion status in CaP with disease aggressiveness [(BCR, pathological Gleason score and Grade Group (GG), prostate specific death) [11–16]. These studies differ by study design, as well as patient clinicopathological features and treatment. Key biological differences are evident across ERG fusion status (positive versus negative), including distinct methylation patterns, with hypermethylation more pronounced in ERG positive versus negative CaP tumors [17, 18]. In addition, the processes of tumor evolution are also different between fusion positive and negative tumors, with *TMPRSS2-ERG* tumors characterized by chromoplexy, while chromothripsis is more common in *TMPRSS2-ERG* negative tumors [19, 20]. Considering the multiclonal and heterogenous nature of CaP, it is important to examine all tumor foci for ERG fusion status as any of these may lead to aggressive CaP [9, 21]. Overall, these findings suggest that tumor etiology is variable, depending on fusion status in CaP.

Based on ERG fusion positive and fusion negative distinctness of CaP, we hypothesized that there may also be differences at the underlying germline level between these two molecular subtypes. CaP is one of the most heritable solid tumors with up to 15% of cases linked to family history [22, 23]. Additionally, inherited germline risk variants have been implicated in different stages of CaP management including screening, staging and treatment [24–26]. Genome wide association studies (GWAS) have identified about 167 common, low penetrance CaP susceptibility variants [27–42]. However, vast majority of GWAS have been performed in populations of European ancestry, only a - few studies are published in men of African-American origin [43–46]. This may have important implications for disease risk prediction across global populations [47], as implied by differences in CaP associated SNPs between AA and CA patients. A whole genome admixture mapping study in AA CaP has identified the 8q24 risk locus to be significantly associated with prostate cancer [48]. We also showed that the Broad11934905 SNP, which segregates with African ancestry, is associated with an increase in non-organ-confined CaP at time of surgery [49].

Thus, it is hypothesized that ERG gene fusion status of AA and CA patients reflects underlying biological and/or genetic differences of CaP development. Since *TMPRSS2-ERG* fusion is considered to be an early event in CaP [50], it is anticipated that SNPs associated with CaP risk may influence ERG fusion status. Therefore, the goal of the present study was to identify germline SNPs associated with ERG status of CaP.

## RESULTS

**Figure 1:**
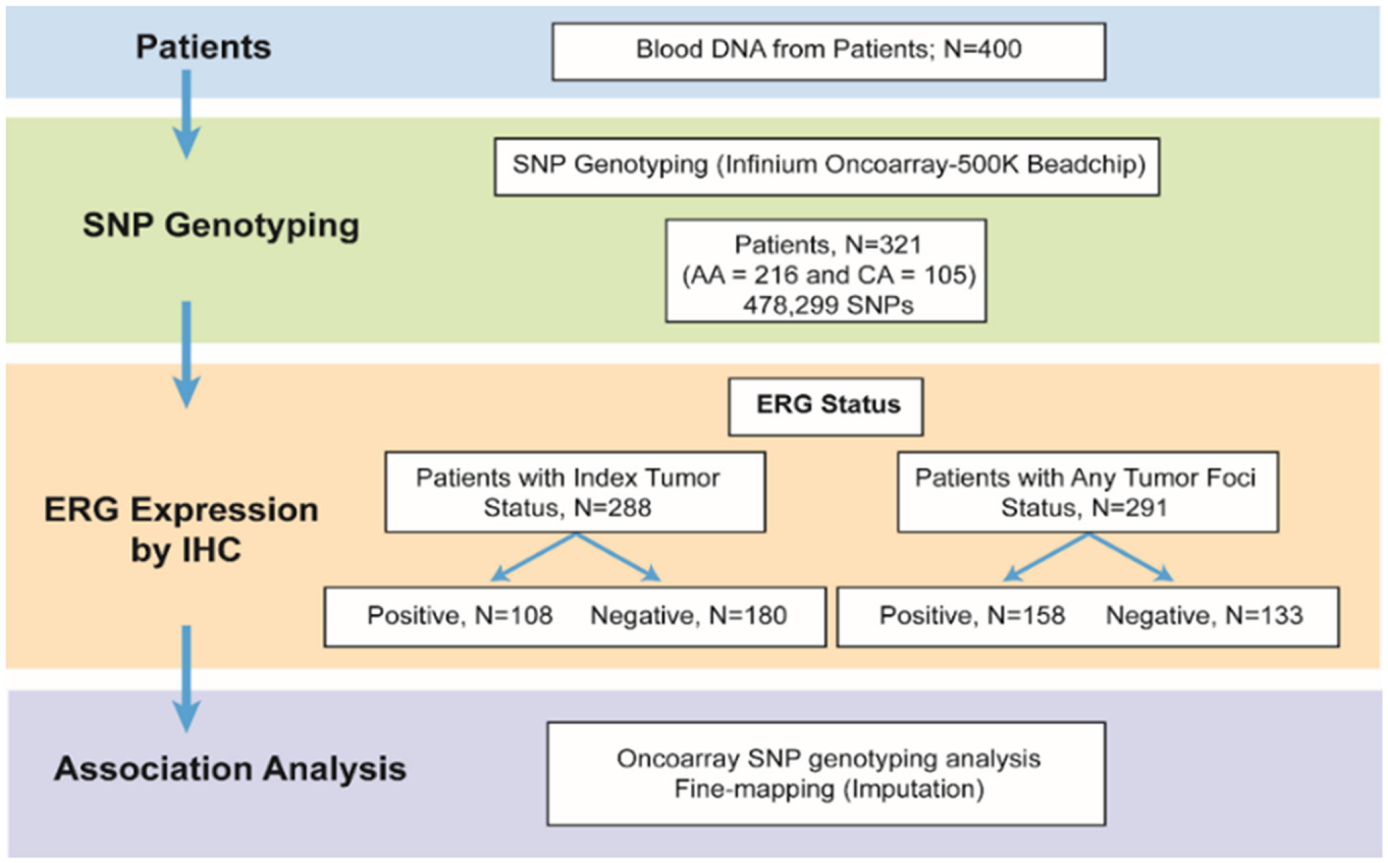
Schematic representation of the study.

The frequency of SNPs on oncoarray in 321 CaP patients was compared between fusion positive and fusion negative CaP subtypes to agnostically examine the association of the inherited variants with *TMPRSS2-ERG* status of CaP, A description of the patients in the study cohort across ERG + vs. ERG - groups is provided in Table 1. Most men had pathological Gleason Grade Group 1–3 tumors and stage pT2. The frequency of ERG positive index tumors was 37.5% (108/288), while the frequency of positive ERG staining in any tumor focus was 54.3% (158/291). Schematic representation of the study workflow is depicted in Figure 1.

**Table 1:** Clinico-pathological characteristics of patients in ERG+ and ERG- prostate cancer

Variable	ERG-	ERG+	*P* value
*N*	182	120	
Age at diagnosis (year)			
Mean (SD)	57.9 (8.4)	56.1 (8.8)	0.0785
PSA at diagnosis (ng/mL)			
Median (range)	5.0 (0.6–129.1)	5.3 (0.5–22.4)	0.6992
FU (year)			
Median (range)	7.5 (0.6–17.3)	7.9 (1.2–17.7)	0.5047
Pathological T stage			
pT2	136 (74.7)	93 (77.5)	
pT3–4	46 (25.3)	27 (22.5)	0.5815
GG			
GG1-3	104 (59.4)	82 (68.9)	
GG4-5	71 (40.6)	37 (31.1)	0.098
Missing			
Surgical margin			
Negative	139 (80.8)	83 (70.9)	
Positive	33 (19.2)	34 (29.1)	0.0509

Abbreviations: N: total numbers; SD: standard deviation; FU: follow-up; pT: pathological T stage; GG: Gleason group. Data represented here is based on index tumor.

### Association between the SNPs and ERG status

For genetic association studies in admixture population, it is important to consider that ancestry differences among the sampled individuals can be a confounder. Failure to appropriately account for population structure due to ancestry admixture can lead to both spurious association (increased type-I error rates—false positive) as well as reduced power (inflated type-II error rates—false negative) (Supplementary Figure 1). To correct for the confounder a variance component approach called Efficient Mixed-Model Association eXpedited (EMMAX) was used (Supplementary Figure 1). This approach is based on pair wise relatedness between individuals, using high-density markers to model the phenotype distribution. EMMAX implements linear mixed model approach for association testing, accounting for global population substructures with an empirical covariance matrix.

**Figure 2:**
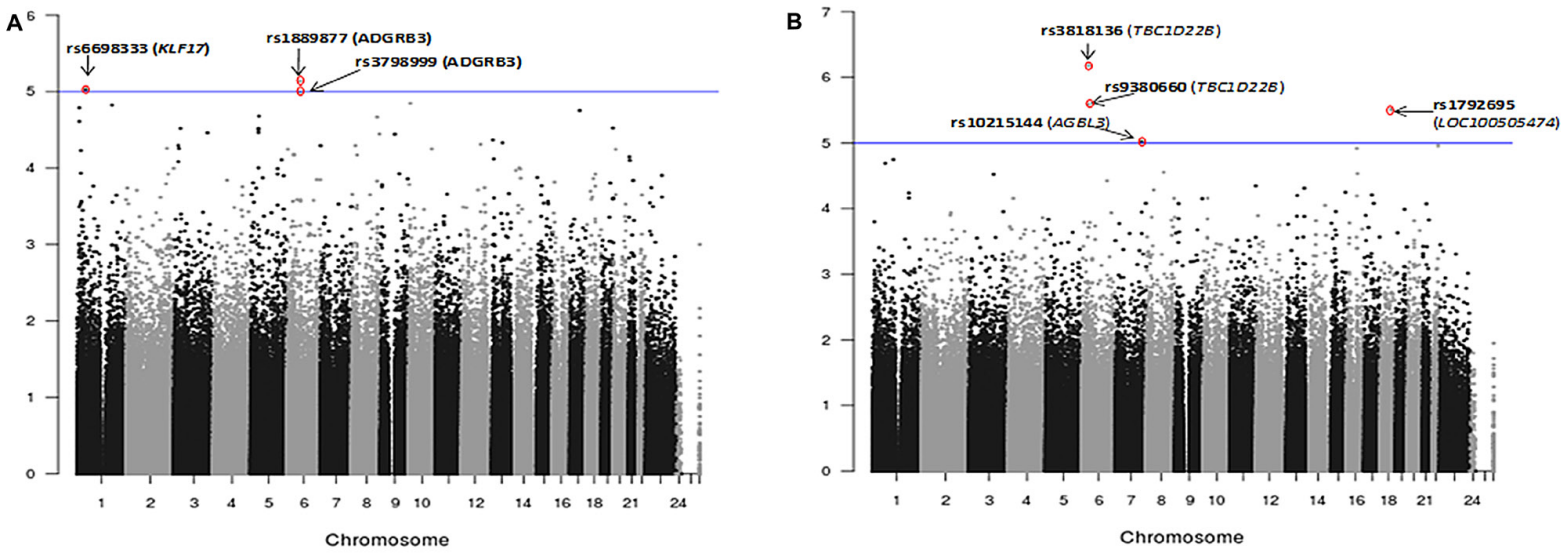
Manhattan plots showing association analysis (EMMAX) of SNPs with (**A**) ERG positive index tumor (*N* = 108) vs. ERG negative index tumor (*N* = 180). (**B**) Any tumor foci positive for ERG (*N* = 158) vs. ERG negative tumor (*N* = 133). A total of 478,299 SNPs are plotted against their respective positions on the chromosomes.

In the EMMAX corrected dataset rs6698333, an intron variant of Kruppel-like factor 17 (*KLF17*) and two SNPs (rs1889877, rs3798999) in the intron of adhesion G protein-coupled receptor B3 (*ADGRB3*) were significantly (< 10^-5^) associated with ERG fusion status of the index tumor (Figure 2; Table 2). The Krüppel-like factor (KLF) family is highly conserved zinc finger transcription factors that regulate cell proliferation, differentiation, apoptosis, and migration. Reduced KLF17 in human cancer affects TGF-β and p53 pathways. *ADGRB3* is a p53-target gene that encodes a brain-specific angiogenesis inhibitor, and is a member of the secretin receptor family.

**Table 2:** Description of the 9 significant SNPs

Chr	SNP	Location	Alleles [minor allele/common allele]	Transcript (s)	Gene (s)	*In-exon*	Mutation (s)	*P*-value	MAF
6	rs1889877	69729678	[A/G]	NM_001704.2	*ADGRB3 (BAI3)*	Intron Variant	NA	0.00549042	0.028
1	rs6698333	44554457	[T/C]	NM_173484.4	*KLF17*	Intron Variant	NA	0.0017769	0.489
6	rs3798999	69714947	[A/G]	NM_001704.2	*ADGRB3 (BAI3)*	Silent	NA	0.00017719	0.486
7	rs10215144	134765148	[A/G]	NM_178563	*AGBL3*	Silent	NA	0.00000952	0.461
6	rs3818136	37252210	[T/C]	NM_017772	*TBC1D22B*	EXON	Synonymous N257N	0.00000067	0.400
6	rs9380660	37305622	[T/G]	NA	NA	NA	NA	0.00000282	0.495
18	rs1792695	53782900	[T/G]	NR_040026 NR_040025	LOC100505474 LOC100505474	Silent, Silent	NA	0.00000319	0.273
6	rs34349373	37254109	-/T	NM_017772	*TBC1D22B*	intron variant	NA	0.00000005	0.419
6	rs2055272	37289781	A/G	NM_017772	*TBC1D22B*	intron variant	NA	0.00000006	0.396

Abbreviations: Chr: chromosome; MAF: minor allele frequency; SNP: single nucleotide polymorphism; NA: not applicable. Location Information is based on genome assembly- GRCh37 (hg19). P value is based on EMMAX analysis using an additive model.

Four SNPs (rs10215144, rs3818136, rs9380660 and rs1792695) were significantly (< 10^-5^) associated with ERG positive phenotype under any tumor focus positive for the fusion (Figure 2, Table 2). rs3818136 is a synonymous variant and rs9380660 is downstream variant of *TBC1D22B*, a GTPase activating protein for Rab family. Rab GTPase proteins are aberrantly expressed in various tumors and are found to be involved in cancer progression. rs10215144 is an intron variant in *AGBL3*, an ATP/GTP binding protein-like 3 and rs1792695 is an intron variant in ncRNA LOC100505474.

We also performed the association analysis for the six known GWAS risk SNPs at 8q24, 5p15, 8p21, 17q24, 19q13 and 22q13 that were reported to be associated with ERG fusion status [51–53]. The present study validates rs11704416 in chromsome 22 and rs16901979 in chromosome 8q24 to be significantly associated with ERG fusion status either by index tumor (rs11704416; *p* = 0.0043; rs16901979; *p* = 0.012) or by any tumor focus positive for ERG fusion (rs11704416; *p* = 0.033; rs16901979; *p* = 0.034) (Supplementary Table 1).

### Genotype imputation analysis

**Figure 3:**
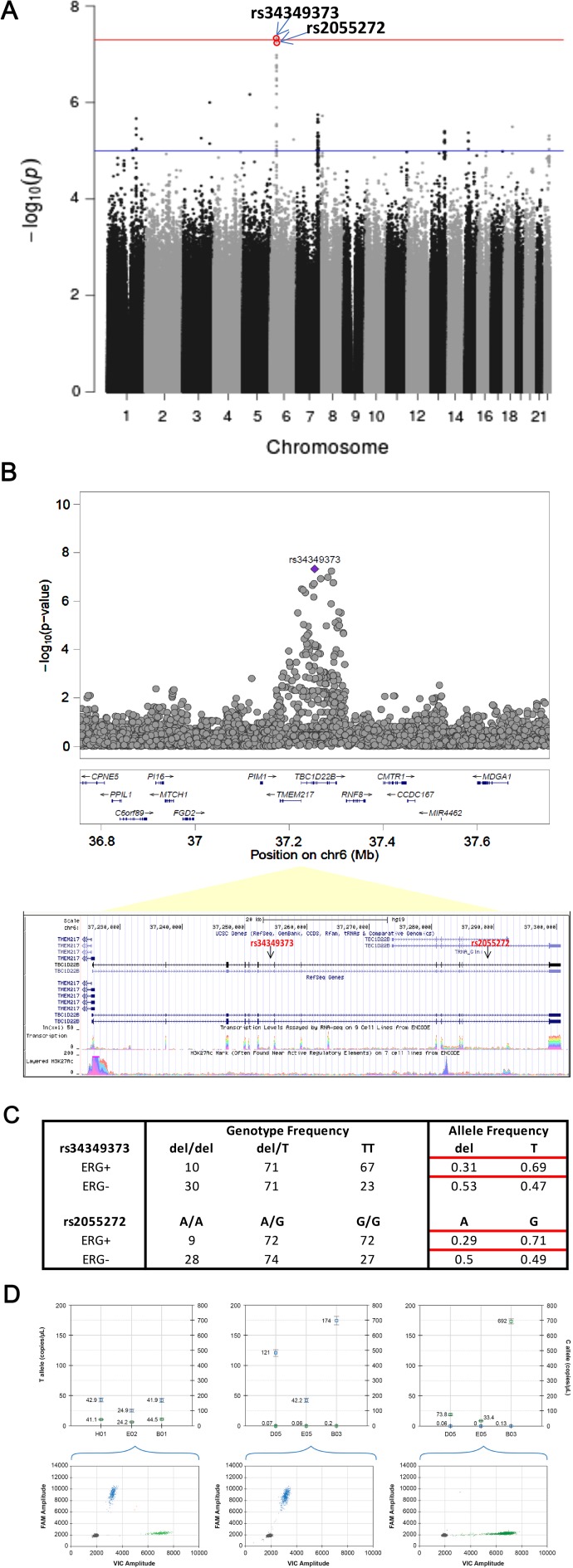
Fine-mapping of genetic associations by imputation analysis (**A**) Imputation analysis (IMPUTE2) of SNPs with any tumor foci positive for ERG phenotype in a total of 158 ERG positive and 133 ERG negative cases. A total of 13 million imputed SNPs with MAF > 1% SNPs are plotted against their respective positions on the chromosomes. (**B**) Plots show association results of imputed SNPs. -log10 *P* values (y axis) of the SNPs are shown according to their chromosomal positions (x axis). The Genome Browser annotation track page zoomed in to display the rs34349373 and rs2055272 (intron variants) in TBC1D22B gene on human chromosome 6, Feb 2009 assembly (hg19) (**C**) Genotype and allele frequencies of the rs34349373 and rs2055272 polymorphisms in ERG positive (by any tumor foci) vs. ERG negative CaP (**D**) Representative graph for SNP genotyping for rs2055272 (C/T) using droplet digital PCR (ddPCR) approach. Upper panel shows concentration (copies/ul) of FAM allele (T; Channel 1) and VIC allele (C; Channel 2) in set of representative samples with 3 genotypes (CT, TT, CC). Lower panel is 2-D Amplitude view where each axis represents the amplitude of fluorescence for either FAM (vertical axis) or VIC (horizontal axis). The FAM probe can hybridize only to the alternate allele (T allele), while the VIC probe hybridizes only to reference allele (C allele).

Imputation analysis of genome-wide Oncoarray (500,000 SNP) data was performed by the IMPUTE2 approach using the 1000 Genomes reference dataset. Imputed SNPs rs34349373 and rs2055272, two intronic variants in *TBC1D22B* (TBC1 Domain Family Member 22B), a GTPase activating protein for Rab family, were significantly (< 10^-6^) associated with ERG positive phenotype in any tumor foci (Figure 3). The 2 variants are found to be in strong linkage disequilibrium in both CA and AA populations with r^2^ of 1.0 and 0.91 respectively. Imputed SNP rs2055272 was further validated by TaqMan based ddPCR genotyping approach. Concordance between Taqman genotypes and imputed genotypes was 98.04% (100/102).

### Association between the SNPs and clinicopathological status

**Figure 4:**
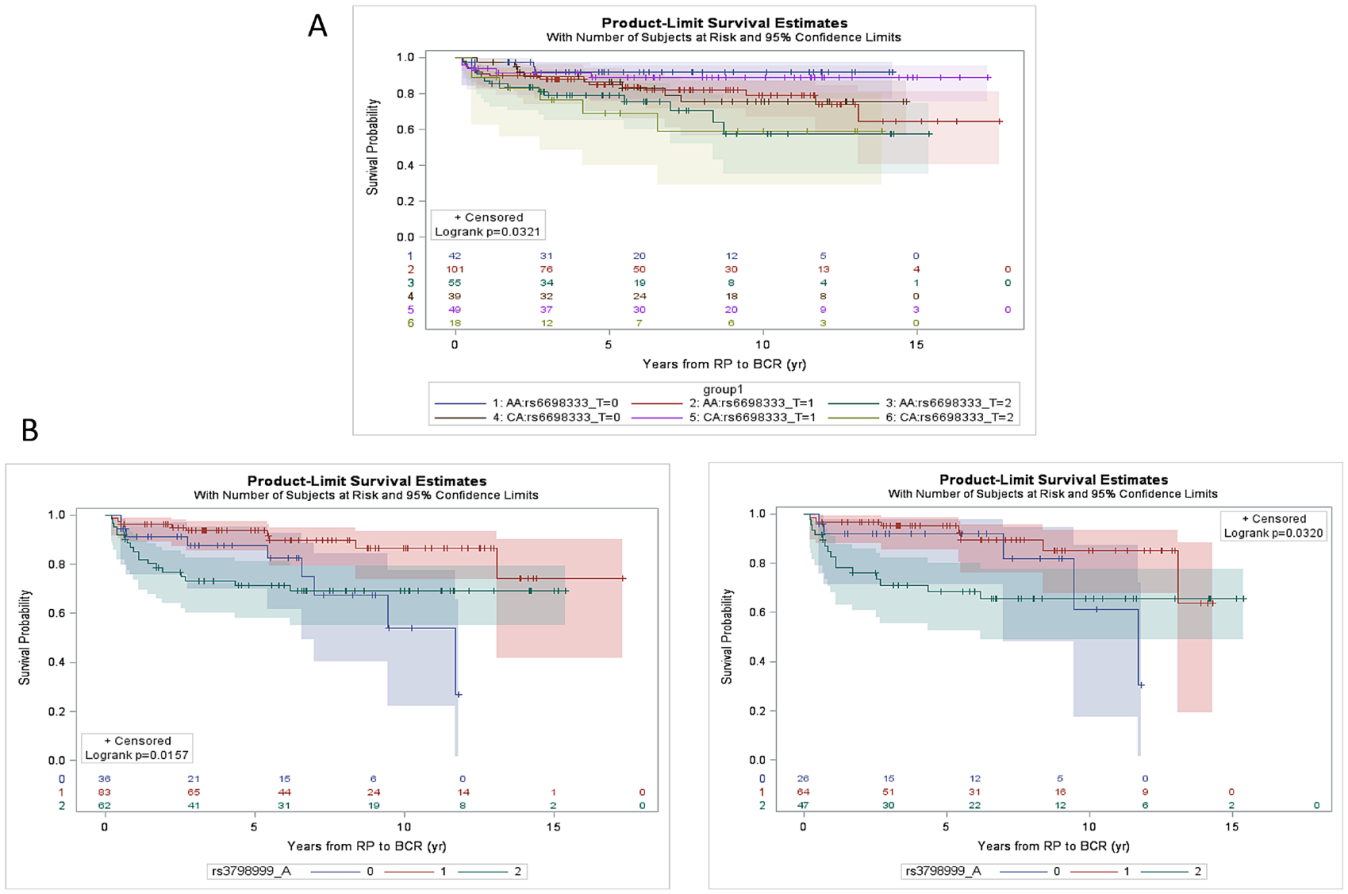
Kaplan-Meier estimation curve of time to BCR as a function of SNPs. (**A**) rs6698333 SNP [Wt (CC), 0 vs Heterozygous (CT), 1 vs Polymorphic homozygous (TT), 2] for CaP patients in AA (*n* = 198) and CA patients (*n* = 106). The log-rank *p* value (*p* = 0.0321) indicates that there is an association between SNP and BCR over time. (**B**) rs3798999 SNP [Wt (GG), 0 vs Heterozygous (GA), 1 vs Polymorphic homozygous (AA), 2] for CaP patients in ERG negative patients (AA and CA combined; *n* = 181; left panel) and for ERG negative AA patients only (*n* = 137; right panel). The SNP was found to be associated with BCR in ERG negative patient cohort (*p* = 0.016) specifically in AA patients (*p* = 0.032).

The 9 SNPs (7 SNPs on Oncoarray chip and 2 imputed SNPs) were assessed for associations with clinicopathologic features, including pathological stage and grade at prostatectomy and incidence of biochemical recurrence. rs34349373 and rs2055272 were significantly (*p* < 0.05) associated with CaP ERG fusion status in both AA and CA patients, where the variant allele is more frequent in ERG negative cases (Supplementary Table 2). However, these SNPs were not associated with pathological stage (pT stage) or Grade Group (GG). Kaplan–Meier analysis indicated no association between imputed SNPs and BCR when stratified by race or ERG status (Supplementary Figure 2). rs3798999 (intron variant in *ADGRB3*) and rs10215144 (intron variant in *AGBL3*) were significantly associated with pT stage (*p* < 0.05) and rs10215144 was also significantly associated (*p* = 0.048) with high grade CaP (GG4-5 vs. GG1-3) on a univariate analysis (Supplementary Table 3). Unadjusted univariate Kaplan–Meier analysis indicated that rs6698333 (intron variant in *KLF17*) was associated with BCR (*p* = 0.032) in both AA and CA patients, where carriers of the risk allele develop BCR significantly earlier during disease progression than the carriers of wild type (wt) allele (Figure 4A).

ERG status based unadjusted univariate analysis revealed that rs3798999 SNP was associated with the development of BCR in ERG negative patients (AA and CA combined; *p* = 0.016) and the association is also present in ERG negative AA patients (*p* = 0.032). The variant shows “protective effect” towards development of BCR (Figure 4B).

## DISCUSSION

This is the first genetic epidemiological study on the association of genetic variants at a genome-wide scale, as opposed to selected SNPs, with *TMPRSS2: ERG* fusion status both by index tumor or by any tumor foci, considering the multifocal and multiclonal nature of the disease [9, 21]. It integrates data on inherited susceptibility and tumor ERG status within a well-defined cohort of men with a median longitudinal follow-up of 7.5 years. Two SNPs were identified: rs34349373 and rs2055272, in *TBC1D22B* on chromosome 6 to be associated with ERG status of CaP, a major driver oncogene in CaP. Minor allele frequency (MAF) reveals that del variant for rs34349373 and A allele variant for rs2055272 are significantly lower in ERG positive cases compared to wild type (wt) in both AA and CA men, thereby implying the association of the SNPs with the fusion negative subtype of CaP. These two SNPs are also in strong linkage disequilibrium (LD; r^2^ > 0.9). After initial validation, it will be important to understand the mechanism by which SNPs influence the formation of fusion protein. rs34349373 (-/T) is an intronic deletion/insertion variation while rs2055272 (A/G) is an intronic SNV in TBC1 domain family, member 22B (*TBC1D22B*) gene with unknown clinical relevance (dbSNP). TBC1D22B is a GTPase activating protein for Rab protein family, and is found to be over-expressed in many cancers.

Comprehensive genetic and epigenetic analyses including our study suggest that tumors with *TMPRSS2-ERG* fusion exhibit different genetic etiology compared to fusion negatives. Similar associations have been observed for GWAS identified genomic loci with the risk of ER-negative disease in breast cancer subtypes [54]. Further, functional analysis based on long range chromatin interactomes analysis in CaP cells has shown strong enrichment of CaP GWAS SNPs at AR-ERG co-binding sites participating in chromatin interactions and gene regulation, suggesting potential functional role of these SNPs towards specific ERG subtype [55].

We hypothesized that SNPs may influence the generation of ERG fusion, which is an early event in CaP carcinogenesis. A genome-wide linkage analysis found that several loci located on chromosomes 9, 18, and X are associated with the development of fusion-positive prostate cancer; however, these studies were performed in familial prostate cancer [56, 57].

There are two studies which focused on candidate SNP approach, covering known GWAS risk variants. The first study, Physicians Health Study (PHS) and Health Professionals Follow-up Study (HPFS), examined 39 known risk variants in a patient cohort of 227 ERG fusion-positive and 260 ERG negative cases [51, 52]. Six SNPs at 8q24, 5p15, 8p21, 17q24, 19q13 and 22q13 were found to be significantly associated with ERG fusion status. The second study by Luedeke *et al.* examined 27 common CaP risk variants using case-case comparison approach on 296 *TMPRSS2: ERG* fusion-positive versus 256 fusion-negative cases, alongwith an independent validation of significant SNPs in a patient cohort of 669 cases. The study found that variants at 8q24 and 17q24 were significantly linked with *TMPRSS2-ERG* fusion status. Interestingly, in both studies, rs1859962 at 17q24 was identified to be significantly associated with fusion positive CaP and the variants in the risk loci of 8q24 were found to be over-represented in fusion negative CaP, implying the role of 8q24 region towards fusion negative subtype of CaP. The present study also replicated 2 of the known ERG associated SNPs (rs11704416 in chromsome 22; rs16901979 in chromosome 8) from PHS and HPFS study. The SNPs were associated with ERG fusion status either by index tumor or by any tumor foci positive for ERG. rs16901979 has been associated with increased risk for CaP in several studies including AA men [36, 58, 59]. SNP rs11704416 was found to be associated with aggressive CaP in a meta-analysis of four GWAS including 5,953 cases of aggressive CaP and 11,463 controls [60].

An earlier study by FitzGerald [16] *et al*. based on association of 5 candidate SNPs in *ERG* and *TMPRSS2* in a cohort of 127 patients, showed a positive association for rs12329760 in chromosome 21 in *TMPRSS2* for fusion subtype resulting from translocation. Another study showed that shorter germline CAG repeat length in *AR* [61] and rare variants in DNA repair genes, *ESCO1* N191S and *POLI* F532S [51] were associated with higher risk of ERG-positive CaP.

However, these studies are mainly based on a few candidate SNPs, in a population predominantly represented by Caucasians. Therefore, it is important to explore the SNP association on a genomewide scale in context of race, as ERG is expressed almost 2 times more frequent in CA than AA CaP. Additionally, in these studies, only one tumor focus was evaluated per specimen which does not take into account focal heterogeneity of the disease. Limitations of the present study includes: a) lack of independent validation b) limited generalizability of the CPDR cohort to other US longitudinal cohorts, as well as RP patients in other nations [7].

In summary, this study identified the association of 2 SNPs (rs34349373 and rs2055272) in *TBC1D22B* with ERG fusion status where the minor alleles are associated with an ERG negative subtype of CaP. Additionally, rs3798999 SNP (*ADGRB3*) was significantly associated with the development of BCR in ERG negative patient cohort. Validation study in independent large patient cohort with race stratified analysis, and functional understanding of the biology of these SNPs in relation to ERG phenotype are warranted. Overall, this study may contribute toward defining the underlying biology and genetics of ERG positive and ERG negative CaP in AA and CA patients.

## MATERIALS AND METHODS

### DNA specimens

In this retrospective cohort-based study, 400 archived genomic DNA specimens were used from blood of CaP patients undergoing radical prostatectomy treatment at Walter Reed National Military Medical Center (WRNMMC) under an IRB approved protocol. DNA was extracted from peripheral blood lymphocytes using Qiagen DNeasy Blood kit. Archived clinicopathological data were evaluated from the 400 patients who self-identified their race as AA or CA.

### *TMPRSS2-ERG* fusion status

ERG status was determined by immuno-histochemistry (IHC) for ERG protein expression, as a surrogate for the *TMPRSS2-ERG* fusion. For ERG IHC analysis, representative whole-mount 4-um cross section encompassing tumor foci with highest grade and/or stage from each prostatectomy specimen were processed and stained with a highly specific anti-ERG monoclonal antibody (clone 9FY; Biocare Medical Inc., Concord, CA, USA) as previously described [8, 62]. The index tumor was identified as the tumor with the largest volume if all foci have the same grade, or with the highest Grade Group. Multiple tumor foci (average no. 5) in representative whole-mount prostate sections were evaluated, per patient, for the presence or absence of the ERG oncoprotein. The patient was called ERG positive when any of the tumor foci was positive. Slide selection, tumor grading, and staining interpretation were performed by a single pathologist.

### SNP genotyping (infinium oncoarray, a 500K BeadChip)

SNP genotyping was performed on the Illumina Golden Gate genotyping platform using Infinium Oncoarray, a 500K BeadChip on a genome wide scale in 400 CaP patients. The oncoarray contains approximately 500,000 SNPs including 275,000 tagSNPs. It includes SNPs covering common ancestry (1,500 SNPs), genetic variants associated with 5 common cancers (breast, colorectal, lung, ovarian and prostate) as well as SNPs covering quantitative traits, pharmacogenetics, and fine mapping of common cancer susceptibility loci. It includes 80,000 prostate cancer specific genetic variants. A total of 496654 SNPs genotype calls were generated from the 400 specimens. Data quality control (QC) was performed by applying sample and SNP QC using PLINK [63]. 14,729 SNPs were excluded due to SNP call rate < 0.90. Additional 1,482 SNPs were excluded due to deviation from Hardy–Weinberg Equilibrium (HWE) test (*P*-value < 1 × 10^-8^). 48 samples were excluded due to sample call rate < 0.95, and 33 samples were further excluded based on plink proportion identity-by-descent (PI_HAT) > 0.15) [64]. The final QCed genotype data included 478,299 SNPs from a total of 321 patients (AA = 216 and CA = 105).

### Statistical analysis

Association analysis was based on Efficient Mixed-Model Association eXpedited (EMMAX) which accounts for population structure, including relatedness between cases [65]. The analysis was performed based on the additive model. Genotype imputation analysis was performed on oncoarray SNP array dataset using IMPUTE2 based on the 1000 Genomes Project data (phase 3) [66, 67]. Imputation analysis was based on a total of 13 million SNPs with MAF > 1%, where we used only well-imputed variants (IMPUTE2 info score ≥ 0.9; MAF >1%). For the initial discovery analysis, we have used lower significant threshold (1 × 10^-5^) to identify possible genetic loci associated ERG fusion. However, for the imputation approach, we have used a higher significant threshold (1 × 10^-8^) to discover probable causal variants associated with ERG fusion. Chi-square testing or Fisher exact test were used to evaluate the associations of genotypes with clinical-pathological variables. Unadjusted Kaplan-Meier survival analysis and log-rank testing were used to show the probability of BCR-free survival stratified by genotypes. All statistical analysis was performed using SAS version 9.4 (North Carolina) and statistical significance was set at *p* < 0.05.

### TaqMan SNP genotyping

Imputation results for rs2055272 were further validated by TaqMan genotyping (assay C_3025729_20, ThermoFisher Scientific) in 102 patients (98.03% concordance). Validation was performed by droplet digital PCR (ddPCR) approach using a QX200 Droplet Generator (BioRad) and the data was analyzed by QuantaSoft software (BioRad). Briefly, a ddPCR mastermix was prepared containing 11 μl 2× ddPCR Supermix (Bio-Rad), 1.1 μl 20× Taqman SNP Genotyping Assay (Applied Biosystems), and 7.9 μl nuclease-free water (Qiagen) per sample. The mastermix was prepared at room temperature and 20 μl was added to 2 μl (5 ng) of each DNA sample. Samples were loaded into individual wells of DG8TM cartridges (BioRad), and droplets were generated using a QX200 Droplet Generator (BioRad). For each sample, 40 μl of droplet mix was then transferred to a 96-well plate, and PCR was performed in a thermal cycler using the following cycling conditions: 95°C × 10 min; 40 cycles of [94°C × 30s, 60°C × 60s]; 98°C × 10s; 40°C × 10 min. The Bio-Rad QX200 Droplet Reader was then used to assess droplets as positive or negative based on fluorescence amplitude. The QuantaSoft software (BioRad) was used to analyze droplet data.

## SUPPLEMENTARY MATERIALS



## Abbreviations

CaP: prostate cancer
RP: radical prostatectomy
DNA: deoxy-ribonucleic acid
SNP: single nucleotide polymorphism
ddPCR: droplet digital PCR
RT-PCR: real-time polymerase chain reaction
LD: linkage disequilibrium
MAF: minor allele frequency
BCR: biochemical recurrence
FFPE: formalin-fixed-paraffin embedded
H&E: hematoxylin and eosin
LCM: laser-capture-microdissection
MAb: monoclonal antibody
