# Supplementary material for: Association of germline genetic variants with TMPRSS2-ERG fusion status in prostate cancer
Source: Oncotarget. 2020 Apr 14;11(15):1321–33. doi: 10.18632/oncotarget.27534 (PMC7170497; doi:10.18632/oncotarget.27534)
Supplement: Supplementary file 1 [file oncotarget-11-1321-s001.pdf]

Association of germline genetic variants with *TMPRSS2-ERG* fusion status in prostate cancer

SUPPLEMENTARY MATERIALS

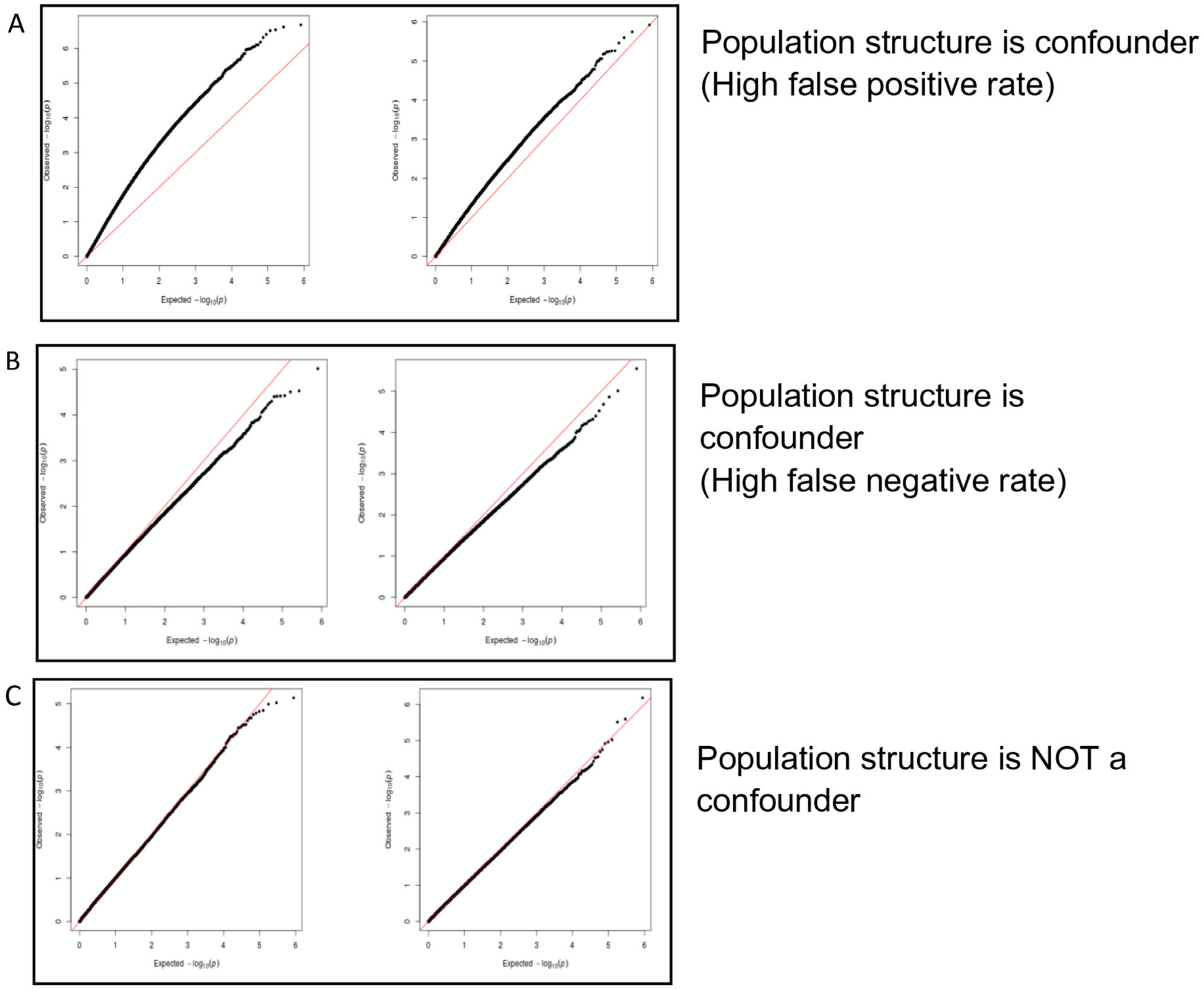

Supplementary Figure 1: Q-Q plots showing expected and observed distribution of associated *p*-values in the ERG fusion positive versus fusion negative by index tumor (Fusion positive, *N* = 108; Fusion negative, *N* = 180; left panel) or any tumor foci positive for ERG (Fusion positive, *N* = 158; Fusion negative, *N* = 133; right panel) using. (**A**) Logistic regression analysis. (**B**) Principal Component Analysis using the EIGENSTRAT software tool. (**C**) Efficient mixed-model association (EMMA) approach.

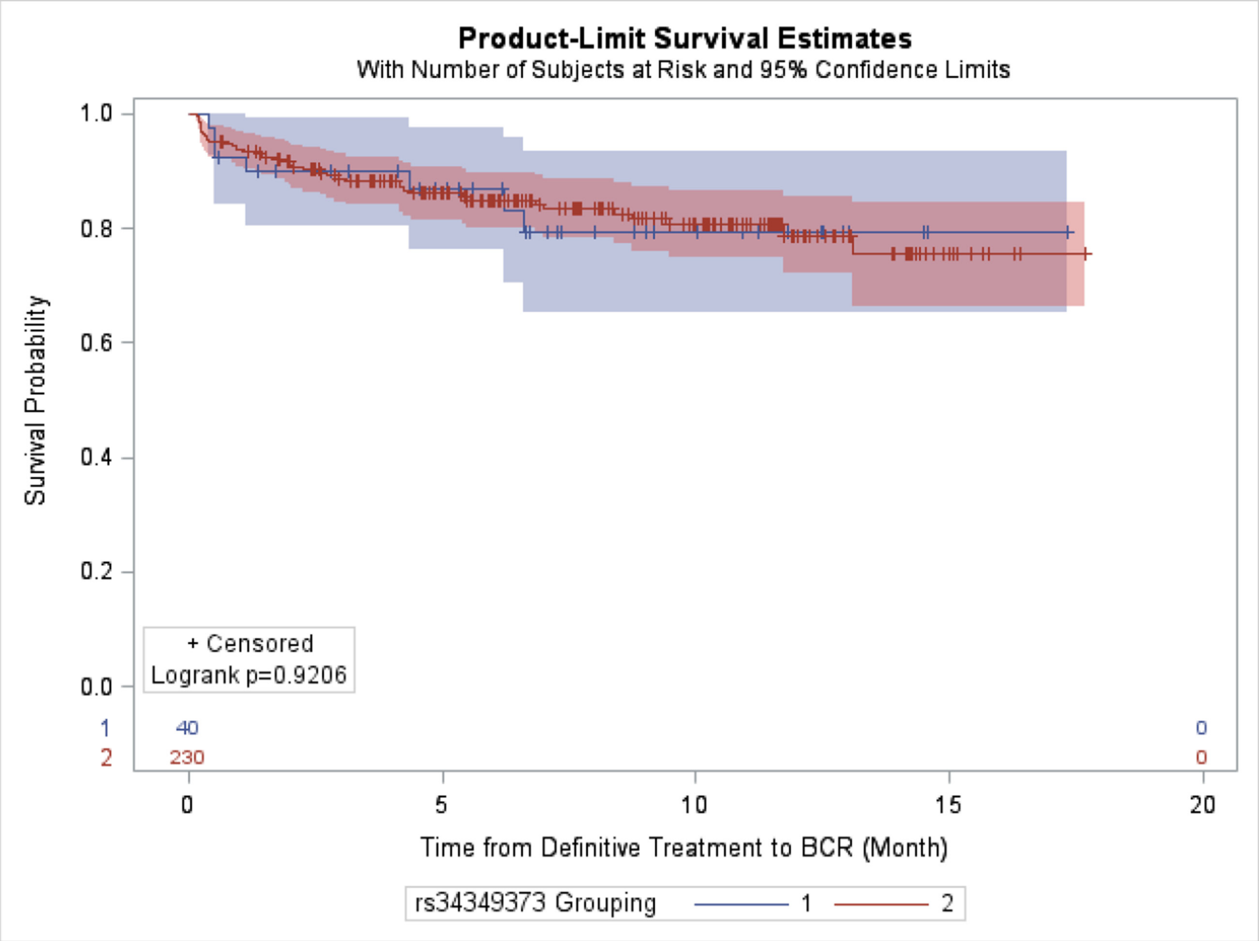

Supplementary Figure 2: Kaplan–Meier estimation curve of time to BCR as a function of rs34349373 (Wt, 11 vs polymorphic homozygous, 22) for CaP patients (*n* = 270). For rs34349373, the log-rank *p* value (*p* = 0.9206) indicates that there no association between SNP and BCR over time.

Supplementary Table 1: Association analysis for 6 known ERG fusion associated CaP SNPs in our dataset

SNP	Chr	Location	MAF	Major allele	Minor allele	Effect allele	Beta (Index Tumor positive for ERG)	P (Index Tumor positive for ERG)	OR (Index Tumor positive for ERG)	Beta (Any tumor foci positive for ERG)	P (Any tumor foci positive for ERG)	OR (Any tumor foci positive for ERG)
rs11672691	19	41985587	0.403	A	G	G	–0.033	0.454	0.9677	–0.0005	0.991	0.9995
rs11704416	22	40436973	0.208	G	C	C	–0.105	0.033	0.9007	–0.1457	0.004	0.8644
rs12653946	5	1895829	0.433	C	T	T	0.060	0.118	1.0620	0.0789	0.048	1.0821
rs1512268	8	23526463	0.402	T	C	C	0.016	0.703	1.0162	–0.0091	0.835	0.9910
rs16901979	8	128124916	0.310	C	A	A	0.101	0.035	1.1068	0.1246	0.012	1.1327
rs1859962	17	69108753	0.369	T	G	T	0.012	0.768	1.0118	0.0120	0.771	1.0121

Abbreviations: Chr: chromosome, MAF: minor allele frequency; SNP: single nucleotide polymorphism; OR: odds ratio. Location Information is based on genome assembly-GRCh37 (hg19). EMMAX analysis was performed based on the additive model. We have included the Odds Ratio in the tables.

Supplementary Table 2: Association of rs34349373 and rs2055272 with ERG status stratified by race

ERG status	Chr	SNP	Location	Counts	OR	ChiSeq	*P*-value
Index_T_AA	6	rs2055272	37289781	180	0.4529	–2.824	0.004741
Index_T_CA	6	rs2055272	37289781	96	0.3493	–3.142	0.001677
Any tumor_foci_AA	6	rs2055272	37289781	185	0.4147	–3.494	0.000476
Any tumor_foci_CA	6	rs2055272	37289781	97	0.2442	–3.748	0.000178
Index_T_AA	6	rs34349373	37254097	170	0.4813	–2.543	0.01099
Index_T_CA	6	rs34349373	37254097	98	0.3904	–2.949	0.00319
Any tumor_foci_AA	6	rs34349373	37254097	175	0.4082	–3.444	0.000574
Any tumor_foci_CA	6	rs34349373	37254097	97	0.2579	–3.778	0.000158

Abbreviations: Chr: chromosome; SNP: single nucleotide polymorphism; OR: odds ratio.

Supplementary Table 3: Association analysis for significant SNPs with clinicopathological features (A) and ERG status (B) of prostate cancer

A

		Pathological T stage			GG		
**SNP**	**Genotype**	**pT2**	**pT3-4**	***P* value**	**GG1-3**	**GG4-5**	***P* value**
	GG	63 (26.9)	18 (24.7)		48 (25.1)	30 (27.8)	
	GA	116 (49.6)	35 (48.0)		96 (50.3)	51 (47.2)	
rs1889877	AA	55 (23.5)	20 (27.4)	0.7853	47 (50.3)	27 (25.0)	0.8525
	CC	65 (27.9)	17 (23.3)		51 (26.8)	28 (25.9)	
	CT	117 (50.2)	34 (46.6)		96 (50.5)	51 (47.2)	
rs6698333	TT	51 (21.9)	22 (30.1)	0.337	43 (22.6)	29 (26.8)	0.7111
	GG	56 (23.9)	32 (43.8)		54 (28.3)	32 (29.6)	
	GA	119 (50.9)	19 (26.0)		92 (48.2)	42 (38.9)	
rs3798999	AA	59 (25.2)	22 (30.1)	**0.0004**	45 (23.6)	34 (31.5)	0.2222
	GG	74 (31.6)	19 (26.0)		59 (30.9)	33 (30.6)	
	GA	114 (48.7)	29 (39.7)		90 (47.1)	48 (44.4)	
rs10215144	AA	46 (19.7)	25 (34.5)	**0.0358**	42 (22.0)	27 (25.0)	0.8268
	CC	80 (34.2)	26 (35.6)		66 (34.6)	38 (35.2)	
	CT	122 (52.1)	38 (52.0)		103 (53.9)	53 (49.1)	
rs3818136	TT	32 (13.7)	9 (12.3)	0.9472	22 (11.5)	17 (15.7)	0.532
	GG	59 (25.3)	22 (30.1)		51 (26.8)	29 (26.8)	
	GT	118 (50.6)	33 (45.2)		98 (51.6)	48 (44.4)	
rs9380660	TT	56 (24.0)	18 (24.7)	0.6607	41 (21.6)	31 (28.7)	0.3392
	GG	125 (53.4)	40 (54.8)		101 (52.9)	60 (55.6)	
	GT	93 (39.7)	27 (37.0)		75 (39.3)	41 (38.0)	
rs1792695	TT	16 (6.8)	6 (8.2)	0.8711	15 (7.8)	7 (6.5)	0.8614

Abbreviations: SNP: single nucleotide polymorphism; pT: pathological T shage; GG- Gleason grade.

Supplementary Table 3: (Continued)
B

SNP	Genotype	ERG-			ERG+		
		GG1-3	GG4-5	*P* value	GG1-3	GG4-5	*P* value
	GG	37 (35.6)	22 (31.0)		11 (13.4)	8 (21.6)	
	GA	47 (45.2)	38 (53.5)		45 (54.9)	13 (35.1)	
rs1889877	AA	20 (19.2)	11 (15.5)	0.549	26 (31.7)	16 (43.2)	0.1303
	CC	17 (16.5)	15 (21.1)		33 (40.2)	13 (35.1)	
	CT	54 (52.4)	31 (43.7)		38 (46.3)	20 (54.0)	
rs6698333	TT	32 (31.1)	25 (35.2)	0.5042	11 (13.4)	4 (10.8)	0.7333
	GG	22 (21.2)	13 (18.3)		31 (37.8)	19 (51.4)	
	GA	49 (47.1)	31 (43.7)		39 (47.6)	11 (29.7)	
rs3798999	AA	33 (31.7)	27 (38.0)	0.6809	12 (14.6)	7 (18.9)	0.1886
	GG	26 (25.0)	28 (39.4)		29 (35.4)	5 (13.5)	
	GA	56 (53.8)	29 (40.8)		33 (40.2)	19 (51.4)	
rs10215144	AA	22 (21.2)	14 (19.7)	0.113	20 (24.4)	13 (35.1)	**0.0487**
	CC	27 (26.0)	22 (31.0)		36 (43.9)	16 (43.2)	
	CT	59 (56.7)	35 (49.3)		42 (51.2)	18 (48.6)	
rs3818136	TT	18 (17.3)	14 (19.7)	0.6223	4 (4.9)	3 (8.1)	0.7834
	GG	20 (19.4)	14 (19.7)		28 (34.2)	15 (40.5)	
	GT	53 (51.5)	31 (43.7)		43 (52.4)	17 (46.0)	
rs9380660	TT	30 (29.1)	26 (36.6)	0.5315	11 (13.4)	5 (13.5)	0.7781
	GG	60 (57.7)	42 (59.2)		38 (46.3)	18 (48.6)	
	GT	38 (36.5)	27 (38.0)		35 (42.7)	14 (37.8)	
rs1792695	TT	6 (5.8)	2 (2.8)	0.6553	9 (11.0)	5 (13.5)	0.8548

Abbreviations: SNP: Single nucleotide polymorphism; GG: Gleason Grade.
